# No Need for Templates in the Auditory Enhancement Effect

**DOI:** 10.1371/journal.pone.0067874

**Published:** 2013-06-27

**Authors:** Samuele Carcagno, Catherine Semal, Laurent Demany

**Affiliations:** Institut de Neurosciences Cognitives et Intégratives d'Aquitaine, Université de Bordeaux and CNRS, Bordeaux, France; Baycrest Hospital, Canada

## Abstract

The audibility of a target tone in a multitone background masker is enhanced by the presentation of a precursor sound consisting of the masker alone. There is evidence that precursor-induced neural adaptation plays a role in this perceptual enhancement. However, the precursor may also be strategically used by listeners as a spectral template of the following masker to better segregate it from the target. In the present study, we tested this hypothesis by measuring the audibility of a target tone in a multitone masker after the presentation of precursors which, in some conditions, were made dissimilar to the masker by gating their components asynchronously. The precursor and the following sound were presented either to the same ear or to opposite ears. In either case, we found no significant difference in the amount of enhancement produced by synchronous and asynchronous precursors. In a second experiment, listeners had to judge whether a synchronous multitone complex contained exactly the same tones as a preceding precursor complex or had one tone less. In this experiment, listeners performed significantly better with synchronous than with asynchronous precursors, showing that asynchronous precursors were poorer perceptual templates of the synchronous multitone complexes. Overall, our findings indicate that precursor-induced auditory enhancement cannot be fully explained by the strategic use of the precursor as a template of the following masker. Our results are consistent with an explanation of enhancement based on selective neural adaptation taking place at a central locus of the auditory system.

## Introduction

Detecting relevant information in noise represents a crucial challenge for sensory systems. One of the strategies that the auditory system uses to achieve this feat is to privilege newly arriving information. For example, a target tone that is added to a multitone background tends to stand out perceptually [1]. This phenomenon, known as auditory enhancement, occurs even if the target-plus-background mixture (test sound) is presented several seconds after the background alone (precursor) [2], indicating that it does not reflect an augmented response to acoustic transients.

One influential hypothesis posits that enhancement results from adaptation processes: When the test sound is presented, neurons tuned to the background frequencies will have been adapted by the previous presentation of the precursor. Therefore, their response will be less strong than the response of the (unadapted) neurons tuned to the added target tone. The differential response of neurons tuned to the background components and neurons tuned to the target component would lead to the perceptual pop-out of the latter [3], [4]. A variant of this hypothesis–the “adaptation of inhibition” hypothesis–states that enhancement results from the decreased inhibition that the background components exert on the target component as a consequence of adaptation [5], [6].

Although there is psychophysical and neurophysiological evidence supporting adaptation-based accounts of enhancement [5]–[7], non-sensory phenomena may also be involved in the phenomenon. Enhancement is often assessed by measuring the increase in the detectability of a target tone turned on synchronously with a background masker, when this mixture is preceded by a precursor consisting of the masker alone. Since common onset time is a powerful grouping factor [8], part of the difficulty in detecting the target tone may stem from difficulties in segregating it from the background masker, even when peripherally they excite different frequency channels. When the precursor is presented before the test sound, it may act as a perceptual “template” of the background masker, helping listeners to identify it and segregate it from the target. This may be especially important when the frequencies of the masker components change from trial to trial.

If precursors are beneficial because they help listeners to identify the background masker, their effectiveness should decrease when they are made perceptually dissimilar from this masker. Adaptation-based interpretations of enhancement, on the other hand, posit that enhancement will be dependent on the spectral relationships between the precursor and masker components, but not on their perceptual similarity *per se*. Regarding this issue, Summerfield et al. [4] found that for a harmonic test sound, the enhancement produced by a harmonic precursor was equivalent to the enhancement produced by a noise precursor with the same spectral envelope. Viemeister et al. [9] also found similar amounts of enhancement for inharmonic stimuli preceded by inharmonic, harmonic, or notched-noise precursors with similar spectral envelopes. These studies suggest that precursor/masker similarity is not crucial for enhancement. However, the authors did not verify that the manipulations applied to the precursor actually reduced its effectiveness as a template of the following masker. It is possible that the precursor, despite being perceptually dissimilar from the following masker within the test sound, was still used effectively by the listeners to identify the masker components and segregate them from the target. Another possible reason why enhancement did not change as a function of precursor/masker similarity in these studies is that the frequencies of the masker components were fixed. In contrast to the studies of Summerfield et al. [4] and Viemeister et al. [9], an experiment by Kidd et al. [10] suggested that precursor/masker similarity plays a role in enhancement. Interestingly, in this experiment, the frequencies of the masker components varied randomly from trial to trial.

Precursor/masker similarity may also play an important role in the enhancement produced by a precursor presented contralaterally to the test sound (i.e., to the opposite ear). This form of enhancement cannot be explained by peripheral adaptation and has been found in studies in which the masker components were randomized from trial to trial [10]–[13], while it has not been found in studies in which the background components were fixed [2], [4], [14]. Therefore, it seems plausible that contralateral enhancement is obtained because the precursor acts as a template of the following masker. Alternatively, contralateral enhancement may result from some form of central neural adaptation.

The aim of the present study was to assess the role played by the perceptual similarity between precursor and masker in ipsilateral and contralateral enhancement. To this end, in a first experiment, we tested whether making the precursor dissimilar from the masker, by gating its components asynchronously, affected enhancement. In a second experiment, we checked that making the precursor asynchronous reduced its effectiveness as a template of the masker.

## General Methods

### Ethics Statement

The two experiments reported here were carried out in accordance with the Code of Ethics of the World Medical Association (Declaration of Helsinki) for experiments involving humans. Both experiments were approved by the Direction Régionale des Affaires Sanitaires et Sociales (Authorization for Biomedical Research N° LR07). All participants gave written informed consent and all, except author SC, were paid an hourly wage.

## Experiment 1

### Listeners

Eight listeners (five males), including author SC, were tested in experiment 1. The listeners ranged in age between 19 and 29 years (mean = 23) and had absolute pure-tone thresholds below 20 dB HL for both ears at octave frequencies from 250 to 8,000 Hz.

### Stimuli and Procedures

**Figure 1:**
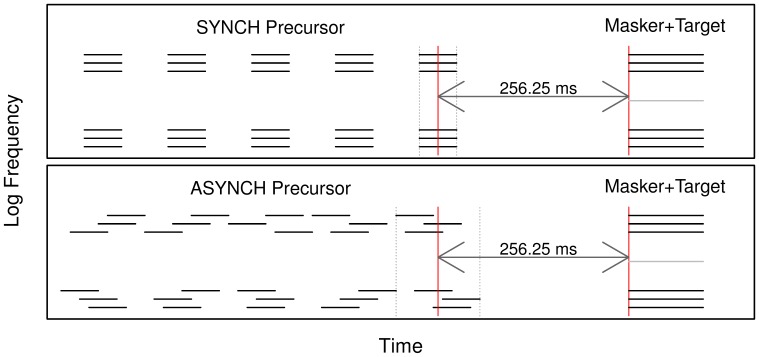
Illustration of the stimuli used in experiment 1. Listeners had to detect a 100-ms target (gray line) embedded in a 100-ms multitone masker (background). The masker-plus-target mixture could be preceded by five bursts of a synchronous precursor (top panel), five bursts of an asynchronous precursor (bottom panel), or silence (not shown). As indicated by the arrows, the time interval between the midpoint of the last precursor burst and the beginning of the masker-plus-target mixture was the same in the SYNCH and ASYNCH conditions.

In each experimental condition, we measured the threshold for detecting a 100-ms target tone presented simultaneously with a multitone masker. A schematic representation of the stimuli used in the experiment is given in Figure 1. On each trial, the target frequency was randomly drawn from a uniform distribution between 600 and 2400 Hz. In order to eliminate uncertainty about the target frequency, a copy of the target was presented at the beginning of each trial, at 50 dB SPL. A sequence of two observation intervals, separated by a 500-ms silent pause, started 500 ms after the offset of this target cue. Each observation interval contained a precursor sound followed by a test sound. The test sound always included a multitone masker; the target tone was added to this masker in only one of the two observation intervals (chosen at random). Listeners had to indicate, by means of a key press on a computer keyboard, whether the target tone was presented in the first or in the second observation interval. The observation intervals were marked by flashing lights on a computer screen and feedback was immediately given after each listener's response through a colored light on the computer screen.

The masker consisted of a lower and an upper frequency band that were composed of three pure tones each, and it was placed symmetrically around the signal frequency. The spacing between the three tones in each masker band was 100 cents (1 cent = 1/1200 octave), while the distance between the target and the masker components closest to it was 350 cents. The level of each masker component was 50 dB SPL. There were three precursor types: SYNCH, ASYNCH and SILENT. In the SYNCH conditions, the precursor was an exact copy of the masker, except that its duration was 50 ms. It was presented five times before the test sound, with a silent inter-stimulus interval (ISI) of 62.5 ms between precursor bursts. In the ASYNCH conditions, the components of each precursor burst, rather than being gated simultaneously, had a 12.5-ms onset asynchrony (see Figure 1); there was no silent interval between the precursor bursts. The order in which the six precursor components were successively gated in an ASYNCH burst varied randomly from burst to burst. For both the SYNCH and the ASYNCH conditions, the time interval between the middle time point of the last precursor burst and the onset of the test sound was 256.25 ms. As a consequence, the ISI between the offset of the last precursor burst and the onset of the test sound was 231.25 ms in the SYNCH conditions and 200 ms in the ASYNCH conditions. In the SILENT conditions, the test sound was separated from the beginning of the observation interval by 793.75 ms of silence. All tones were gated on and off with 10-ms raised-cosine ramps.

In the SYNCH and ASYNCH conditions, the precursor was presented either to the same ear as the test sound (“*Ipsi”* condition) or to the opposite ear (“*Contra”* condition), while the initial target cue was always presented to the same ear as the test sound. In order to investigate possible effects of frequency region, we used three interleaved adaptive tracks estimating thresholds separately in a LOW (600–952 Hz), a MID (952–1512 Hz) and a HIGH (1512–2400 Hz) frequency region. For each track, the target level was initially set at 60 dB SPL and was varied adaptively following a 2-down 1-up rule tracking the 70.7% correct point on the psychometric function [15]. The step size was 4 dB for the first four reversals and 2 dB thereafter. Track selection was pseudo-random, with a maximum of three consecutive trials per track permitted. A block of trials was terminated when at least 12 reversals per track had occurred. If, in a given track, the total number of reversals was even, the threshold for that track was computed as the average of all the reversals after the 4^th^; otherwise, the 5^th^ reversal was also discarded.

Listeners completed twelve sessions. During each session, they completed one block of trials for each precursor type (SILENT, *Ipsi* SYNCH, *Contra* SYNCH, *Ipsi* ASYNCH and *Contra* ASYNCH); these five blocks were randomly ordered. The first two sessions were considered as practice sessions, and the final thresholds were computed as the arithmetic average of the remaining ten threshold estimates, for each precursor type and frequency region.

Listeners were seated in a double-walled sound attenuating booth (Gisol, Bordeaux). The stimuli were generated digitally in Python with 32-bit resolution and a 48-kHz sampling rate on a computer housed outside the booth. They were played through a 24-bit digital-to-analog converter (RME Hammerfall DSP Multiface) and presented via TDH-39 headphones fitted with audiocups that ensured no interaural cross-talk at the presentation levels we used.

### Results and Discussion

**Figure 2:**
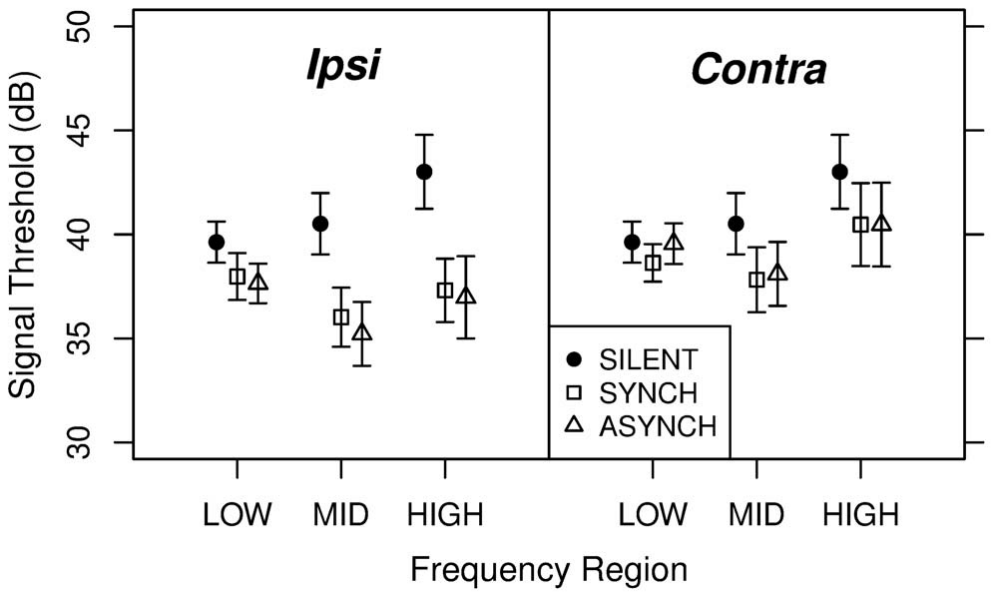
Average target thresholds in experiment 1 (±1 s.e.). The left and the right panels show respectively the thresholds for detecting the target in the *Ipsi* and in the *Contra* conditions, for the SYNCH and ASYNCH precursor type. For the SILENT precursor type, the obtained thresholds are plotted in both panels.

**Figure 3:**
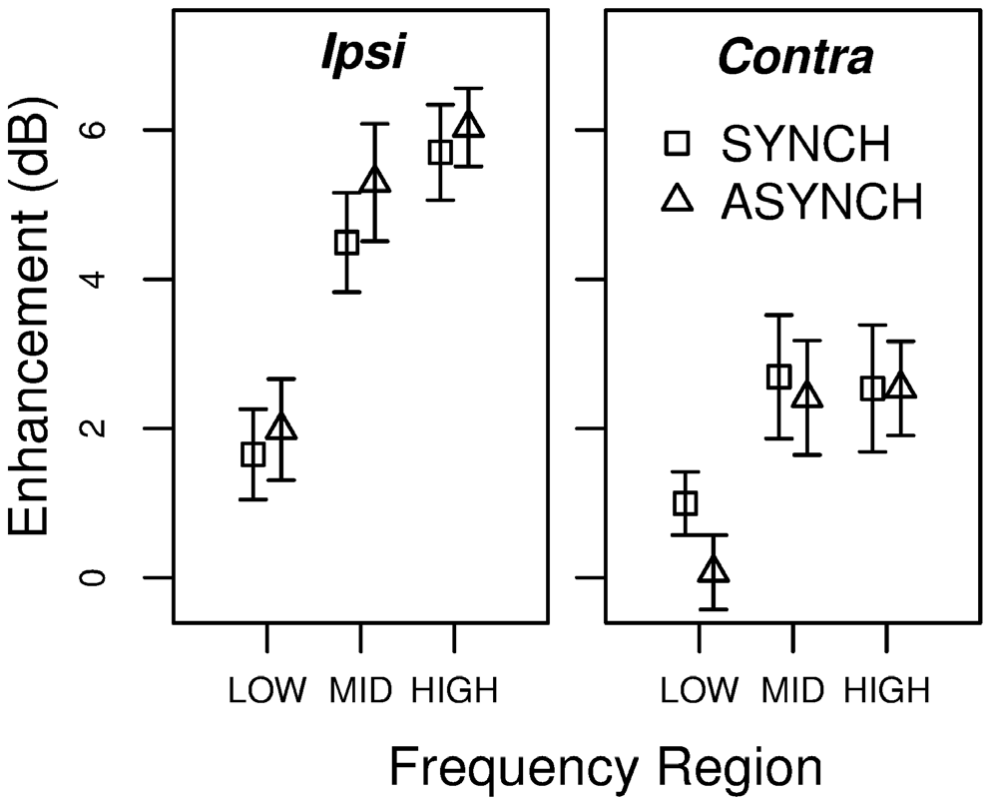
Average enhancement magnitude in experiment 1 (±1 s.e.). The figure displays enhancement magnitude as a function of precursor laterality (*Ipsi* vs. *Contra*) and frequency region.

The average target threshold for each experimental condition is plotted in Figure 2. Figure 3 displays enhancement magnitude, defined as the difference in threshold between a given condition with a non-silent precursor and the corresponding SILENT condition. Overall, enhancement magnitude was about 4 dB in the *Ipsi* case and 2 dB in the *Contra* case. Averaged across frequency regions, enhancement was significantly greater than zero for each precursor type [*Ipsi* SYNCH: *t*(7) = 8.66, *p*<0.001; *Ipsi* ASYNCH: *t*(7) = 9.08, *p*<0.001; C*ontra* SYNCH: *t*(7) = 4.65, *p* = 0.002; *Contra* ASYNCH: *t*(7) = 3.72, *p* = 0.007]. Thus, some enhancement was obtained even when the precursor and the following masker were dissimilar and/or presented to opposite ears.

The enhancement data were entered in a repeated-measures analysis of variance (ANOVA) with laterality (*Ipsi* vs. *Contra*), synchronicity (SYNCH vs. ASYNCH) and frequency region (LOW, MID, or HIGH) as within-subject factors. The ANOVA revealed a significant main effect of laterality [*F*(1, 7) = 52.5, *p*<0.001] and frequency region [*F*(2, 7) = 10.24, *p* = 0.002], but no main effect of synchronicity [*F*(1, 7) = 0.01, *p* = 0.918]. The interaction between laterality and synchronicity was significant [*F*(1, 7) = 9.64, *p* = 0.017], as well as the interaction between laterality and frequency region [*F*(2, 14) = 14.56, *p*<0.001], while the other interactions were not significant. These results indicate that enhancement was overall stronger in the *Ipsi* than in the *Contra* conditions, and that the effects of synchronicity and frequency region were dependent on the laterality factor.

In order to investigate these interactions, we performed separate ANOVAs for the *Ipsi* and *Contra* conditions. For the *Ipsi* conditions, there was no significant effect of synchronicity [*F*(1,7) = 0.87, *p* = 0.382], while the effect of frequency region was highly significant [*F*(2, 7) = 18.45, *p*<0.001]. The interaction between synchronicity and frequency region was not significant [*F*(2,14) = 0.29, *p* = 0.750]. Follow-up *t*-tests (corrected with the Holm procedure [16]) indicate that enhancement was significantly weaker in the LOW frequency region than in both the MID [*t*(7) = −4.90, *p* = 0.003] and the HIGH [*t*(7) = −5.35, *p* = 0.003] frequency regions, which did not significantly differ from each other [*t*(7) = −1.39, p = 0.206]. In the *Contra* conditions, as in the *Ipsi* conditions, there was no main effect of synchronicity [*F*(1,7) = 1.43, *p* = 0.270] and no significant interaction of synchronicity and frequency region [*F*(2,14) = 0.87, *p* = 0.441], but a significant main effect of frequency region [*F*(2,14) = 4.34, *p* = 0.034]. Follow-up *t*-tests indicate that enhancement was significantly weaker in the LOW region than in the MID region [*t*(7) = −3.22, *p* = 0.044]; the other differences were not significant (LOW vs HIGH [*t*(7) = −2.49, p = 0.083]; MID vs HIGH [t(7) = 0.02, p = 0.987]). Overall, similar patterns of results were obtained in the *Ipsi* and *Contra* conditions, despite the significant interactions found in the main ANOVA. The significant interaction between laterality and synchronicity reflects the fact that whereas in the *Ipsi* conditions there was a trend for greater enhancement with asynchronous than with synchronous precursors, the opposite was true in the *Contra* conditions.

## Experiment 2

### Rationale

The results of experiment 1 indicate that perceptual similarity between the precursor and the following background masker is not crucial to obtain enhancement. However, it could be hypothesized that the asynchronous precursors, despite their dissimilarity, were nonetheless efficient spectral templates of the background masker. In other words, it is possible that listeners could exploit the asynchronous precursors as efficiently as the synchronous precursors to help them identify the components of the masker. The aim of experiment 2 was to test this hypothesis. Whereas, in experiment 1, the listeners' task was to detect the *addition* of a tone to a copy of the precursor, the task in experiment 2 was to detect the *subtraction* of a tone from a copy of the precursor. In the latter task, enhancement could play no role [17]. We reasoned that if, contrary to our assumption, synchronous and asynchronous precursors could be used equally well as templates of the following background masker in experiment 1, then they should also be equivalent in experiment 2.

### Listeners

Twelve listeners (eight males) were tested in experiment 2. Seven of these listeners, including author SC, had also taken part in experiment 1. The listeners ranged in age between 19 and 43 years (mean = 24), and had absolute pure-tone thresholds below 20 dB HL for both ears at octave frequencies from 250 to 8,000 Hz.

### Stimuli and Procedures

As in experiment 1, listeners were presented with five bursts of a synchronous or an asynchronous precursor, followed by a synchronous test sound. However, each precursor burst now had the same frequency components as the masker-plus-target mixtures of experiment 1; therefore each precursor burst now had seven frequency components. The following test sound either had the same seven components or did not contain the central one. The precursor and test sounds were transposed in frequency from trial to trial according to the same rule used in experiment 1; therefore, the frequency of the central precursor component was again drawn from a uniform distribution ranging from 600 to 2400 Hz. On each trial, a single precursor-test sequence was presented and listeners had to judge whether the precursor and test contained the same frequency components or not. Feedback was provided at the end of each trial, as in experiment 1.

Again, synchronous and asynchronous precursors were used in different blocks of trials. However, all stimuli were now presented diotically. The duration of each precursor and test sound component was the same as in experiment 1. Given that each precursor burst now had seven components, its duration in the ASYNCH condition was 12.5-ms longer than in experiment 1. Therefore, the inter-burst ISI in the SYNCH condition was also increased by 12.5 ms. In the ASYNCH condition, the ISI between the offset of the last precursor component and the onset of the test sound was 200 ms. This ISI was set to 237.5 ms in the SYNCH condition, so as to equalize, for the two conditions, the time interval between the midpoint of the last precursor burst and the onset of the test sound. The central component of the precursor and of the test sound (when the test sound had seven components) had the same intensity level; this level was adjusted for each listener in a preliminary phase of the experiment so as to avoid floor or ceiling effects (mean dB SPL = 47.6, sd = 4). The level of all the other precursor and test sound components was set to 50 dB SPL. The preliminary adjustment phase served also as training and lasted about one hour for the listeners who had taken part in experiment 1. The listeners who had not taken part in experiment 1 were given an additional hour of training. After the preliminary phase, listeners completed in a single one-hour session eight blocks of 50 trials for each of the two conditions (SYNCH and ASYNCH). These 16 blocks of trials were randomly ordered.

### Results

The average *d'* in the SYNCH condition was 2.2, while in the ASYNCH condition it was 1.1. This difference was statistically significant [*t*(11) = 4.68, *p*<0.001]. Eleven out of the twelve listeners performed better in the SYNCH than in the ASYNCH condition; the remaining listener showed only a weak trend in the opposite direction. This outcome suggests that synchronicity had a more important effect in experiment 2 than in experiment 1. In order to test the significance of this difference, we converted the *d*' values obtained in experiment 2 to *z*-scores and similarly we converted the enhancement magnitudes measured in experiment 1 to *z*-scores. We then took the differences of the *z*-scores between the SYNCH and ASYNCH conditions for each experiment and compared their means. This test revealed that synchronicity had a significantly greater effect in experiment 2 than in either the *Ipsi* [*t*(18) = 3.81, *p* = 0.001] or the *Contra* [*t*(18) = 2.93, *p* = 0.009] condition of experiment 1. In other words, synchronous precursors gave a significantly greater performance advantage (compared to asynchronous precursors) in experiment 2 than in experiment 1. This result implies that the lack of a significant effect of synchronicity in experiment 1 cannot be explained by assuming that synchronous and asynchronous precursors were equally effective templates of the following masker.

## Discussion

In experiment 1, we found that for test sounds consisting of synchronous components, enhancement effects of similar magnitude were produced by precursor sounds consisting of synchronous or asynchronous components. On the other hand, experiment 2 showed that when enhancement was not involved, it was more difficult to compare the frequency contents of asynchronous and synchronous sounds than to compare the frequency contents of two synchronous sounds. The latter result implies that the asynchronous precursors employed in experiment 1 were less efficient spectral templates of the following maskers. Overall, our results indicate that enhancement cannot be fully explained as a consequence of the precursor acting as a spectral template of the following background masker, which would aid listeners in segregating the target from the masker. This conclusion is in line with the results of two previous studies that found enhancement using precursors that were perceptually dissimilar from the following masker [4], [9]. However, none of these studies had verified that making the precursor perceptually dissimilar from the masker actually reduced its effectiveness as a spectral template of the masker.

Kidd et al. [10] also addressed the issue of precursor/masker similarity. These authors used a task requiring the identification of melodic patterns embedded in multitone maskers. On each trial, before the melodic patterns to be identified, listeners were presented either with an exact copy of the multitone masker or with a notched-noise band that covered the same frequency range and had the same overall level. The authors found that while the notched-noise band improved performance relative to a baseline condition with no precursor, it was less effective than an exact copy of the multitone masker. The results of Kidd et al., therefore, suggest that precursor/masker similarity may have an influence on enhancement. Although we did not find evidence for this in the current study, our results are not incompatible with this hypothesis. What our study shows is that, even if precursor/masker similarity may play a role in certain circumstances, it cannot fully explain either ipsilateral or contralateral enhancement. The reason why Kidd et al., unlike us, observed an effect of precursor/masker similarity in their study may be that perceptually segregating the melodic target from the background was a major difficulty in their task, while in our signal detection task the major difficulty was to detect the tonal target despite the inhibition that the masker exerted on it. It should also be pointed out that in the study of Kidd et al., the masker and target frequencies were drawn from a frequency range (∼5 octaves) which was larger than in our study (2 octaves). The greater masker uncertainty in the study of Kidd et al. increased the potential usefulness of the precursors as masker templates.

It has been proposed that high envelope correlations between the precursor components cause them to be grouped together, forming a stream against which it becomes easy to detect an added component [14]. This “grouping” explanation of the enhancement phenomenon seems a priori unlikely in our experimental conditions, for two reasons. First, the precursor and the masker were separated by a relatively large silent interval. Second, in some conditions they were also presented to different ears. These two factors are clearly unfavorable to the sequential grouping of the precursor and masker components into a single stream [18]. In addition, the grouping hypothesis is in much the same difficulty as the template hypothesis in accounting for the combined results of experiments 1 and 2. Given that the correlation between the precursor components was lower in the ASYNCH condition, the grouping hypothesis predicted worse thresholds in that condition than in the SYNCH condition. This prediction was not confirmed by the results of experiment 1. It could be argued that the asynchrony was not sufficient to affect grouping. However, manipulating the synchronicity of the precursor components did have a large effect in experiment 2. It would be hard to explain why grouping should play a role in experiment 2 but not in experiment 1.

In the current study, we found that the magnitude of enhancement was greater when the target sound was presented above about 1 kHz than when it was presented in a lower frequency region. The origin of this frequency effect is unclear. It has been hypothesized that enhancement reflects activation of the medial olivo-cochlear efferent reflex (MOCR) by the precursor sound [19]: The MOCR would cause a frequency-specific reduction in the gain of the cochlear amplifier, thus decreasing the ability of the background components to mask the target component. There is evidence that the cochlear amplifier has a weaker action at low frequencies than at high frequencies [20]. Thus, the MOCR hypothesis could account for the increase in enhancement that we observed in the higher frequency regions. However, several recent studies that have investigated the frequency tuning of the MOCR in humans using otoacoustic emissions have failed to provide much evidence that the MOCR is strictly frequency specific [21]–[25]. Therefore, it is unlikely that activation of the MOCR can explain the enhancement effects that we report here.

Several authors have hypothesized that enhancement is due to precursor-induced adaptation of neurons tuned to the background components. Viemeister and Bacon [5] found that enhancing a target tone increases its forward masking of a subsequent signal. This finding, replicated several times [6], [26], [27], indicates that exposure to the precursor causes an increase in the effective level of the target tone. This increase in the effective level of the target tone has been interpreted as the consequence of a reduction in the inhibition that the adapted background components would exert on the target tone (adaptation of inhibition hypothesis). Our results are fully consistent with adaptation-based accounts of enhancement. If these accounts are correct, the fact that we found significant enhancement for contralateral precursors implies that at least part of the precursor-induced adaptation producing enhancement takes place centrally, at a point where the monaural auditory pathways have already converged. Neurons showing enhanced responses to a target tone in a multitone background, after the presentation of a precursor consisting of the background alone, have been found by Nelson and Young [7] in the central nucleus of the inferior colliculus (CNIC). The effect of presenting the precursor ipsilaterally or contralaterally to the target-plus-background was not investigated in that study. Given that the CNIC contains neurons showing excitatory responses to both ipsilateral and contralateral ear stimulation [28], [29], it is plausible that some of the neurons found by Nelson and Young show enhanced responses also when the precursor is presented contralaterally to the target-plus-background. The fact that some CNIC neurons show excitatory responses for stimulation of one ear and inhibitory or null responses for stimulation of the other ear may explain why the magnitude of ipsilateral enhancement is greater than the magnitude of contralateral enhancement.
